# Preparation of Green Low Strength Mixture for Foundation Reinforcement Treatment by Using Fly Ash and Waste Coal Gangue

**DOI:** 10.3390/ma13030664

**Published:** 2020-02-02

**Authors:** Linhao Li, Guangcheng Long, Kunlin Ma, Hongwei Ma, Wenbing Wang, Cheng Zhang, Youjun Xie

**Affiliations:** 1 School of Civil Engineering, Central South University, Changsha 410075, China; llhmoshou@163.com (L.L.); xieyj@csu.edu.cn (Y.X.); 2 Electrification Bureau (Zhengzhou), China Railway Signal & Communication Co., Ltd., Zhengzhou 450000, China; agl999@126.com (H.M.); zzzhongyuantielu@126.com (W.W.); zygsjskfb@163.com (C.Z.)

**Keywords:** fly ash coal gangue mixture, mechanical properties, stress–strain curve, durability, greenness index

## Abstract

Effective foundation reinforcement treatment is essential for modern large and complex infrastructure, while it is significant for developing new green high-performance materials for foundation reinforcement. This study investigates a new green concrete by using high volume fly-ash and coal gangue aggregates, which is expected to apply for foundation treatment of modern infrastructure with high loading-bear ability. In this experiment, 12 mix proportions of fly ash coal gangue mixture (the material name, abbreviated FGM) were designed, and its mechanical properties and durability performance were investigated. The mechanical properties of FGM include compressive strength, dynamic elastic modulus, dynamic shear modulus, Poisson’s ratio, and the stress–strain behaviors. The durability performance was evaluated by the parameters of acid resistance, which simulated an acid circumstance. After that, the environmental effects about carbon emission of this material were also investigated. Results show that the FGM with 84.6% wastes utilizing rate is a cost-effective material for foundation reinforcing treatment. Its compressive strength at 28 days and 60 days can reach more than 8 MPa and 10 MPa, respectively. After being immersed in the acid environment for 140 days, the mass loss (%) of the material could be under 3.5%. The greenness shows that the e-CO_2_ indices of FGM are lower than 20 kg/MPa·m^3^, and the e-energy indices are at below 150 MJ/MPa·m^3^. FGM has the advantages of acid resistance, waste recycling, and lower carbon emissions than the previous methods for foundation improvement.

## 1. Introduction

The foundation improvement can uniformly disperse surface load into the original soft soil foundation and effectively reduce railway settlement [1]. With much more strict requirements for high-speed railway construction, the technology for ground improvement has been being developed: (I) At the first stage, lime and cement were used to improve marine clay [2,3]; (II) Then, water glass and other chemical grouting materials could be injected with arbitrary curved intrusion pass into soft foundation to enhance properties [4]; (III) In the third stage, cement-fly ash-gravel mixture (CFG) with a compressive strength of more than 20 MPa was introduced in foundation improvement for high load-bearing infrastructure [5]. CFG materials can not only enhance the subgrade (to the extent of more than 10 MPa), but also provide an opportunity to consume industrial waste solid (fly-ash), which will reduce the engineering cost by more than 50% and promote environmental protection after comparing with former ways [6]. Afterwards, Xiao et al. [7] proposed an optimum design of CFG for soft ground. Recently, because of the shortage of natural sand and limestone [8], utilizing recycle aggregates or solid wastes can be confirmed to replace the concrete’s gravel or sand [9]. It has been recognized as an economical and environmentally friendly method that brings many social benefits.

Coal gangue, a well-known solid waste and a by-product originated from coal production [10], is widely stored in the northwest of China [11]. On the basis of many case studies, it has been widely applied in cement-based material [12]. Although the utilization rates of coal gangue in 2013 could reach about 64% [13], this level could not be defined as “high-efficiency” compared with other waste utilization [14]. Nowadays, a large number of new methods for utilizing coal gangue [10,15,16,17] have been introduced and applied, such as road backfill, farmland drainage, and even roadbed material in express highways. These methods can avoid secondary environmental pollution to air, underground water, and underground soil, unlike the previous treatments that lead to pollution with sulfur dioxide, arsenic, and mercury. Therefore, for the green sustainable development, a new green fly ash coal gangue mixture (a material that abbreviated called FGM) for foundation improvement was designed and researched in this study. It can simultaneously recycle two solid wastes (coal gangue and fly-ash) and mostly reduce construction costs. According to the results [9,18], although its coarse aggregate is comprised totally of coal gangue, the 28 d strength value of coal gangue concrete will still be at 14.7–34.3 MPa. This value can partly meet high load-bearing requirement for some large infrastructure construction [19]. With a large amount of construction material consumption and exploitation, it is worth consideing utilization of waste of coal gangue and fly ash to prepare appropriate foundation reinforcement materials. 

In this study, a new green material with high volumes of fly ash and coal gangue (FGM) was designed to meet the higher load-bearing requirements of foundations [20] in some critical infrastructure, such as high-speed railway. Its mechanical properties and the durability were investigated in indoor experiments. The mechanical properties include compressive strength, elastic modulus, shear modulus, and stress–strain curve. The durability was assessed by acid resistance. Acid resistance is a vital ability for long-term foundation, because the underground water is acid, and the ambient soil in the regional soft foundation can product acid corrosion. It can be explained that the plant species were responsible for the pH of 0–1 in edaphic variables associated with soil depth [21]. To make a simulation for acid underground, the FGMs were designed to be soaked with acid solution and water (as control group), separately. The acid resistance was performed by indexes of elastic modulus loss, weight loss, and strength loss changing during corrosion. Some of the test results from Zhou [22] were also selected as reference to investigate the improvement and development in FGM. Additionally, high energy consumption and carbon emissions were considered as the most significant environmental effects in concrete preparation [23]. Consequently, the greenness of FGM for atmosphere should also be studied to determine its environmental value. To investigate the environmental impacts of FGM, two indices (e-CO_2_ index, CI; e-energy index, EI) [24] were introduced in this research. By considering a combination of the environmental effects and the engineering properties (cubic compressive strength), the two indices were obtained and demonstrated in Equations (1,2),

(1) $$\mathrm{CI}=\frac{\mathrm{embodied}\;-{\mathrm{CO}}_{2}(\mathrm{kg}/m^{3})}{\sigma (\mathrm{MPa})}$$

(2) $$EI=\frac{\mathrm{embodied}\;-\mathrm{energy}(MJ/m^{3})}{\sigma (\mathrm{MPa})}$$

where embodied-CO_2_ represents how much CO_2_ exhausted from preparing unit m^3^ concrete; embodied-energy represents how much energy consumed from producing unit m^3^ concrete; σ is the 28 days compressive strength of the concrete. The latest research showed the embodied environmental impact of self-compacting concrete (SCC) [24] and ultra-high performance concrete (UHPC) [23]. Based on the above analysis and methods, the greenness analysis of unit FGM was assessed by the indices. Additionally, FGM mixtures with different proportions were designed, which is aimed at researching how the parameters—such as water–cement ratio, aggregate gradations, binder amount, fly-ash proportion, and types of coal gangues—affect the properties and indices. Finally, suggestions for FGM preparation and application in future projects are provided. All results in this study can be regarded as the guidelines or references, based on the engineering requirements, to develop clean techniques in high-quality foundation treatment. 

## 2. Experimental Details

### 2.1. Raw Materials

**Figure 1:**
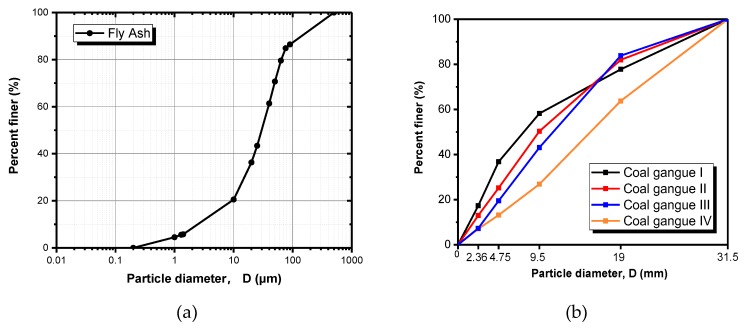
Grading curves of fly ash and coal gangue. (**a**) Fly ash; (**b**) Coal gangue.

The raw materials used in this experiment include class F fly ash, Portland cement, and four different coal gangues. Fly ash is from Xiangtan Power Plant, Xiangtan, China, and its grading curve is presented in Figure 1a. Ordinary Portland cement should be selected for future widespread application in real engineering. The ASTM Type I cement is chose, and it was produced by China United Cement Corporation, Beijing, China. Coal gangues, originated from four different places (Xuchang, Taiyuan, Changzhi, Yulin), were supplied by Lu’an Group Guozhuang Coal Industry Co., Ltd, Changzhi, China. After coal gangues are crushed by coal cracker, their size distribution curve is presented in Figure 1b. In this research, coal gangue from Xuchang is named coal gangue I; coal gangue from Taiyuan is named coal gangue II. Coal gangue from Changzhi is named coal gangue III. Coal gangue from Yulin is named coal gangue IV. The mineral analysis [25,26] on coal gangue and fly ash were done by the same authors from this article. It can be demonstrated that coal gangue has quartz (SiO_2_), mullite (3Al_2_O_3_·2SiO_2_), hematite (FeCO_3_), calcite (CaCO_3_), feldspar (KAlSi_3_O_8_). For fly ash, quartz (SiO_2_), quicklime (CaO), hematite (FeCO_3_), and mullite (3Al_2_O_3_·2SiO_2_) could be found. The chemical compositions percentage and ignition loss (IL) of the raw materials are shown in Table 1. The chemical compositions were tested by X-ray fluorescence with elemental analysis and chemical analysis; IL of the materials was tested in a temperature-controlled furnace for a set time. After a certain cooling, the mass of tested ones was redetermined.

### 2.2. Mix Design and Specimen Preparation

The design of mix in FGM was referenced with the previous work of Zhou [22] and be modified for meeting some requirements, attaching a better performance and new character investigated. Firstly, considering the indoor experiments rather than cast in-situ, the FGM should be ensured its feasibility and meet the demand for casting well and easily. Hence, the water to binder ratio (w/b) was more than 0.5 to ensure the flowability of fresh concrete. The percentage of fine coal gangue (size below 4.75 mm) was below 33.4%, while the percentage of coarse coal gangue (size between 4.75–31.5 mm) was at the range of 58–78%. The ratio of fine to coarse aggregate were designed according to the “8-18” band gradation theory [27]. The theory indicates that the aggregate system consisting of 8 units fine aggregate and 18 units coarse aggregate possesses a optimum aggregate particle gradation and thus benefits for the target properties of mixture in fresh and hardened state. To reduce the cement consuming in FGM production, the fly ash was employed to replace cement at the level of 40–60% of total binder volume. The percentages of the binder in FGM accounts for 11–13% of mass. In all groups, there are no water reducing agent added, and details of the groups are presented in Table 2.

The specimens were prepared in two kinds of size: 72 prisms with 100 mm × 100 mm × 300 mm and 108 cubes with 100 mm × 100 mm × 100 mm. To obtain every result point, there are 2–3 repeated test before calculating the average values. For mixtures, dry ingredients (cement, coal gangue, fly-ash) were firstly mixed by a vibratory concrete mixer with double-horizontal shafts for 2 min until water added, and then were mixed for extra 3 min before fresh concrete being cast into the molds. After 24 h, the specimens were demolded from the molds and stored into the standard-curing room (20 ± 2 °C of temperature and over 98% of relative humidity) with covering plastic membrane, to keep the moisture. They were not taken out until being used for the experiments.

### 2.3. Experimental Methods

#### 2.3.1. Compressive Strength

The 108 specimens at a size of 100 mm × 100 mm × 100 mm were subjected to unconfined compressive strength test by complying with ASTM C-39 [28] after 7, 28, and 60 days. The chosen of the day was aimed at seeing the strength evaluation with curing age in different groups, because of geopolymer effects from coal gangue on long term strength of concrete [29]. The strength results were also taken into discussions about their relationships to the corresponding elastic modulus by fitting the power functions that have been proposed in the articles [30,31]. The prediction model about their relationship was built in this study. The influence of types of coal gangue, amount of cementitious materials, aggregate gradation, water to cement ratio, and fly ash proportion on mechanical performance was also regarded as the parameters to be studied. 

#### 2.3.2. Dynamic Elastic/Shear Modulus

At the curing ages of 28 days and 60 days, and the dynamic elastic modulus and the dynamic shear modulus of the specimens were measured by Young’s modulus tester equipment according to ASTM C-469 [32]. The sizes of the 24 tested specimens were uniformly 100 mm × 100 mm × 300 mm, and the Poisson’s ratio (ν ) of each specimen was obtained by the Equation (3) [33]

(3) $$\nu =\frac{E}{2G}-1$$

where *E* represents specimen’s dynamic elastic modulus (GPa), and *G* represents specimen’s dynamic shear modulus (GPa)

#### 2.3.3. Stress–Strain Curve Test

The above 24 specimens were also used for stress–strain cueve test to evaluate the constitute mode of FGM, following the modulus tests. The stress–strain curve tests were conducted on the 60 days, by using INSTRON 1346 compression machine. When each specimen was tested, a high-precision laser displacement sensor was mounted to the load plate to measure its longitudinal displacement, and the data of stress during loading process was recorded by a computer that is connected with the machine from first to last. When the strain percentage reached 0.5% or the load stress reached a constant steady value, the loading process would be stopped.

#### 2.3.4. Durability Test

On considering the actual circumstance with acid water, it is determined that hydrogen ion (H^+^) plays an important role in concrete corroded. Therefore, in this test, the acid solution was composed of nitric acid and water with a pH of 1–2 for simulating an actual corrosive environment [34]. The other acids—such as hydrochloric or sulphuric acids—were not selected, because of their chloric (Cl^−^) and sulfate (SO_4_^2−^) ions that could simultaneously deteriorate mortar [35,36]. The concentration of the acid solution was weekly re-measured and regulated for stable 1–2 pH value. Next, control groups (only immersed in water without any acid) were set to make comparison. In this test, each mix used four 100 × 100 × 300 mm specimens, which were cured for 28 days. The specimens were respectively immersed in the acid solution (24 ones) and water (as control group of 24 ones) for 140 days. The evolutions of mass-loss rate and modulus-loss rate were gradually shown by recording the statistics after specimens immersed for 28, 56, 84, 112, and 140 days. After that, the damaged specimens were subjected to the test of uni-axial compressive strength by complying with norms ASTM C-39 [28] to gain the strength-loss rate. To keep the acid solution original and expel other contaiminants in the solution, the solution was replaced with a new one in the interval of every month on schedule. The modulus loss rate was calculated according to Equation (4)

(4) $$\Delta E_{\mathrm{ni}}=\frac{E_{ai}-E_{wi}}{E_{wi}}\times 100\%$$

where $\Delta E_{\mathrm{ni}}$ represents modulus loss rate of specimens that had been immersed in acid solution and water immersion for *n* days; $E_{wi}$ is dynamic elastic modulus (GPa) of specimen that had been immersed in water for *i* days; $E_{ai}$ is dynamic elastic modulus (GPa) of specimen that had been immersed in acid solution for i days. Based on GBT/50082-2009 (Chinese Standard for test methods of long-term performance and durability of ordinary concrete), the mass loss rate can be calculated according to Equation (5)

(5) $$\Delta M_{ni}=\frac{M_{ai}-M_{0i}}{M_{0i}}\times 100\%$$

where $\Delta M_{ni}$ represents the mass loss rate of specimen that had been immersed in acid solution for *n* days; $M_{0i}$ is the mass (kg) of specimen that would be corroded by acid solution; $M_{ai}$ is the mass (kg) of specimen that had been immersed in acid solution for n days. Finally, the strength loss rate can be calculated according to Equation (6)

(6) $$\Delta \sigma_{i}=\frac{\sigma_{w}-\sigma_{a}}{\sigma_{w}}\times 100\%$$

where $\Delta \sigma_{i}$ represents the strength loss rate of specimens that had been immersed in acid solution for 140 days; $\sigma_{w}$ means the axial compressive strength (MPa) of specimen that had been immersed in water for 140 days; $\sigma_{a}$ means the axial compressive strength (MPa) of specimen that had been immersed in acid solution for 140 days.

#### 2.3.5. Green Degree Indices of FGM

As we all know, due to the increasing awareness of sustainable development in industrialization, the environmental impact of concrete is an essential factor to be considerated for life cycle assessment approach [24]. In this research, the two indices of CI, EI were used as green degree indices to assess the environmental impact of FGM. The most of raw materials in FGM were fly-ash and coal gangue, which were generally treated as the industrial wastes [37], so utilization rates of waste were high (more 84.6% of total weight), and resources consumption index (evaluated by RI) was not necessary to be considered in this research. For the elements of Equations (1) and (2), σ is the compressive strength of FGM at 60 days. Additionally, embodied CO_2_ emissions (e-CO_2_) and embodied energy consumption (e-energy) of each group were calculated by summing up the products of e-CO_2_ and e-energy of each raw material and the unit volume weight of materials in FGM. The values of e-CO_2_ and e-energy from cement and fly ash were referred by the researches [23,24], and the values of four different coal gangues were provided by CRSC (Zhengzhou) Electrification Bureau Co, Ltd., (Zhengzhou, China) [38]. The embodied carbon dioxide and the embodied energy consumption of coal gangues were generated in the coal gangue processing (including sieving and crushing), and this research did not consider factors of storage and transportation. Meanwhile, limestone was normally used the traditional aggregate, and its values are also provided by the research [39,40]. Based on the foundations of the above report and studies, the e-CO_2_ and e-energy of the raw materials are shown in Table 3. It can be found that cement makes a huge difference in energy consumption and discharge of CO_2_, since cement production needs extraction and furnace burning. The e-CO_2_ and the e-energy of each group were presented in Table 2. 

## 3. Results and Discussion

### 3.1. Compressive Strength

**Figure 2:**
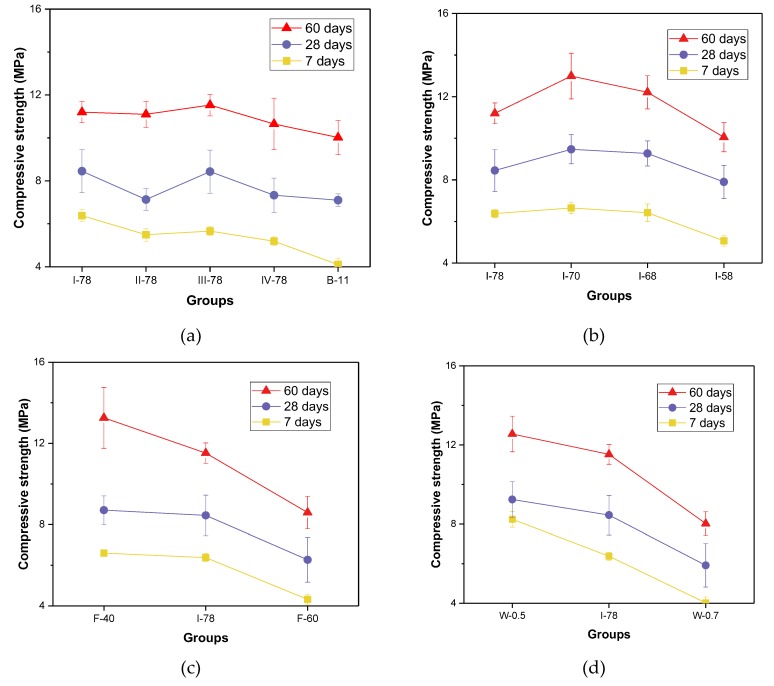
Results of compressive strength for different FGM specimens. (**a**) Coal gangue types and binder volume effect; (**b**) Aggregate gradation effect; (**c**) Fly ash proportion effect; (**d**) Water to binder ratio effect.

The compressive strength results of all specimens in different curing ages are displayed in Figure 2. As shown as Figure 2a, the strength of FGM after 28 days (I-78, III-78 groups) could reach more than 8 MPa, while other different types of coal gangue groups have the strength of more than 7 MPa (II-78 and IV-78). As we all know, the strength of concrete is dependent upon the strength of hardened matrix and the interfacial zone property of respective aggregate [41,42]. The coal gangues in this research originate from different places and have respective features like chemical compositions and particle size distribution. These affected mixture strength and lead to strength variation. Therefore, how to find a suitable way to select the coal gangue that has high strength could be investigated in future research. It has much of significance for practical engineering. Decreasing the total binder proportion could diminish the strength at 28-day age by 16%. The lower binder amount means the fewer content of hydrated silicate calcium gel, calcium hydro-oxide crystals, and ettringite formed, which contribute to the strength development [43]. With the coarse aggregate accounting for 58–78% of total mass, the strength value showed a parabolic variation tendency (see in Figure 2b). Typically, with the coarse aggregate makes up around 70% in total aggregate (called “8-18” band gradation of aggregate) [27], the concrete strength can reach the maximum value. The I-70 has the “8-18” band aggregate gradation, and its strength is more than 9 MPa at 28 days and over 12MPa after 60-day age.

As shown in Figure 2c, increasing the fly ash to cement ratio can decrease the strength. Intriguingly, when the percentage of fly ash ranges from 50% to 60%, the strength value of mixture reduces dramatically. In F-40 group, the strength was improved a lot after 28 days (from 8.71 MPa to 13.26 MPa). In the combination of fly ash and cement, the binder system is actually alkaline. Under the alkaline condition, the fly ash was active thoroughly during the later period, and the secondary cement production also made contributions to the strength after 60 days [44]. The other two groups with less cement could not be observed this improvement. Conventionally, the dramatic fall in strength with increasing water to binder ratio can also be found in Figure 2d. It could be explained that the binder system of FGM was diluted by water added, and then, the contribution to strength from unit binder was reduced. However, the strength after 28 days could still sustain more than 6 MPa when the w/b reached at 0.7.

### 3.2. Dynamic Modulus and Dynamic Poisson’s Ratio

Generally, dynamic elastic modulus reflects the strain of the material responding to the dynamic stress for certain specimen. Dynamic shear modulus describes the shear strain of the material which is resulted from the shear stress. Poisson’s ratio reflects the orthogonal strain of the material, under the uniaxial load. These three properties are the important engineering properties of concrete and can be used to evaluate the deflection of structures for concrete service in practical engineering. The two dynamic modulus values of different FGM are listed in Table 4, together with the Poisson’s ratios obtained by Equation (3). It can be seen that the Poisson’s ratio will not see significant change with the curing age. However, reducing the binder proportion will result in the big boost of the ratio, from 0.510 to 0.787. Besides, different types of coal gangue mixed by the same proportion can also produce diverse Poisson’s ratio in FGM. Their Poisson’s ratio could spread over the range of 0.51–0.6, after 28 days curing.

**Figure 3:**
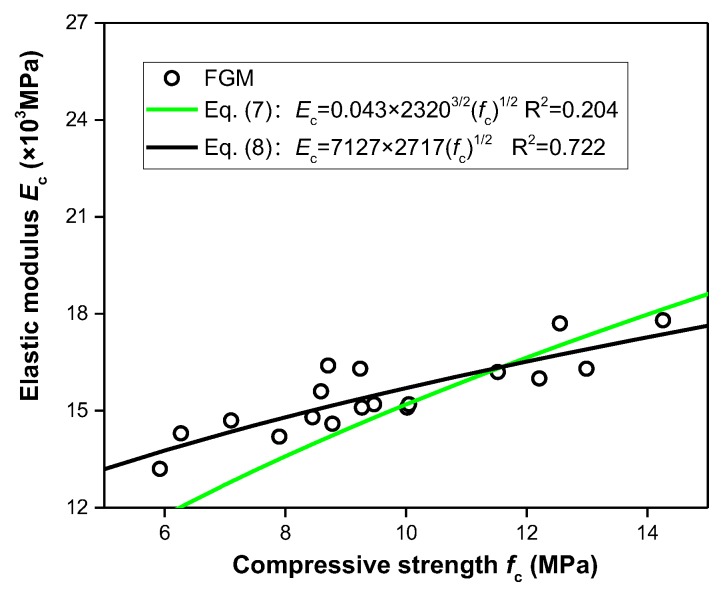
Relationship between compressive strength and elastic modulus.

**Figure 4:**
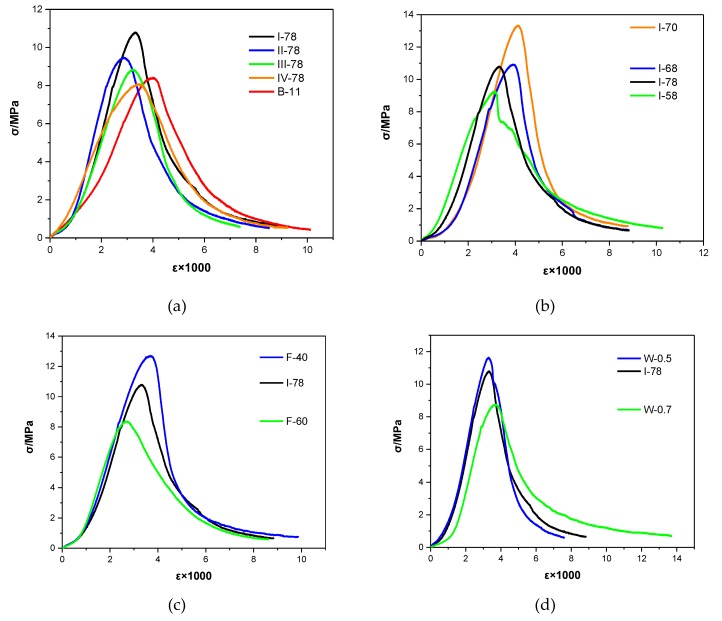
Comparison of stress–strain curves in different FGM specimens. (**a**) Coal gangue types and binder volume effect; (**b**) Aggregate gradation effect; (**c**) Fly ash proportion effect; (**d**) Water to binder ratio effect.

The dynamic elastic modulus values corresponding compressive strength at the same time were plotted on the graph Figure 3 as the dots. According to the previous resutls [30,31], the relationship between Young’s modulus of elasticity *E*_c_ (×10^3^ MPa) and compressive strength *f*_c_ (MPa) can be illustrated by the power function of Equation (7), which are normally suitable for fitting under the strength requirement of 0–20 MPa. The element *w*_c_ in Equation (7) presents the unit weight (kg/m^3^) and is 2320 in this study. The function Equation (7) is displayed in Figure 3; however, the dots are not regularly situated on its curve, with its calculated R^2^ coeffecient of only 0.204. On the other perspective, based on the formulation model of this function, the relationship between elastic modulus and compressive strength of FGM could be illustrated by the regression analyses of Noushini [45] and be built as Equation (8). With Equation (8) shown as a curve in Figure 4, it can be easily found that the dots are nearly situated on the curve, and the correlation coefficient (R^2^ = 0.722) is more than 0.7.

(7) $$E_{c}=0.043w_{c}{}^{3/2}{\left(f_{c}\right)}^{1/2}$$

(8) $$E_{c}=7127+2717{\left(f_{c}\right)}^{1/2}$$

### 3.3. Stress–Strain Relationship

Figure 4 presents the stress–strain curves of different FGM and shows how the curves were influenced by the related variables in this research. In Figure 4a, the slopes of the ascending segment among the four curves (I\II\III\IV-78) were similar, and B-11 ascending segment looks less steep. Compared with other groups, the peak strain of B-11 was significant higher. Because of lateral unconfinement to the specimens and the comparatively higher Poisson’s ratio [46], the transverse strain of B-11 were larger and developed quickly. However, this did not occur in coal gangue types, which could not make much effect on stress–strain performance of mixture. On the next, the slope of descending branch can indicate the failure mode of concrete: moderate descending indicates the ductile damage model, and steep descending indicates the brittle damage model [47]. The F-60 group curve in Figure 4c has a relatively moderate descending branch, which is different from other four curves. Therefore, increasing fly-ash proportion in total binder will form the ductile damaged model in FGM, which is a good performance for alleviating the high velocity impact from the dynamic load. 

For stress–strain curve, considering the similar geometrical character emerged in the normal concrete [48], this research adapts Guo’s model to fit with the curves in FGM. The model function includes Equations (9) with the ascending and descending branch,

(9) $$\begin{array}{l} y=\left\{ \begin{array}{lll} ax+(3-2a)x^{2}+(a-2)x^{3} & ,x\leq 1 & \left(a\right)\\ \frac{x}{b{\left(x-1\right)}^{2}+x} & ,x\geq 1\; & \left(b\right) \end{array}\right.\\ x=\varepsilon /\varepsilon_{c}\;,\;y=\sigma /\sigma_{c} \end{array}$$

In these equations, ε is the strain of the stress–strain curve. $\varepsilon_{c}$ is the value of the strain corresponding the peak stress. σ is the stress and $\sigma_{c}$ is the peak stress in the curve. The parameters of a and b can are determined in ascending and descending branches of the curve, respectively. Based on the models fitted with the curves, the parameter a and the parameter b were calculated and shown in Table 5, as well as the corresponding regression coefficient (R^2^). The all of regression coefficients are higher than 0.94, which can demonstrate that the Guo’s model is suitable for establishing the constitute model of FGM. The damage character and the stress–strain relationship of FGM investigated in this study are essential information for deeper mechanical analysis. The extra tasks need to be done to investigate the prediction of FGM stress–strain curve that based on future vast database from practical engineering. 

### 3.4. Resistance to Acid Corrosion

#### 3.4.1. Appearance of Corroded Specimens

**Figure 5:**
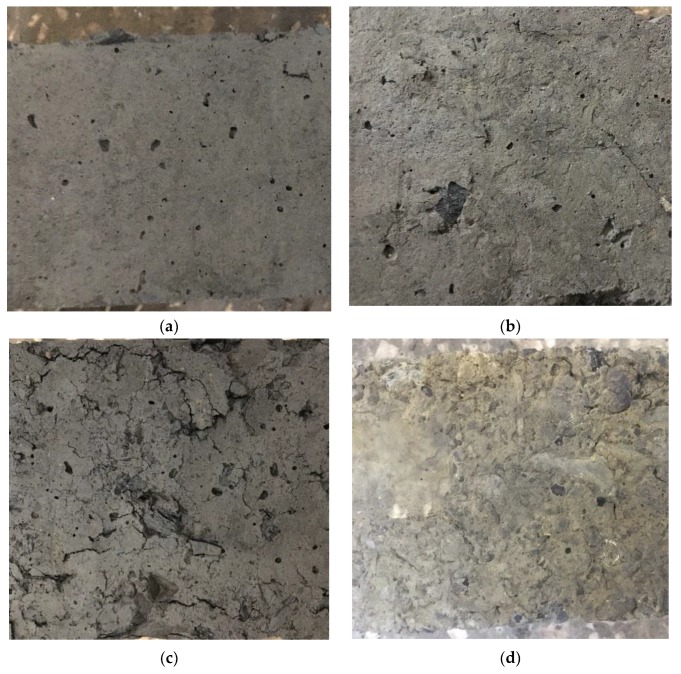

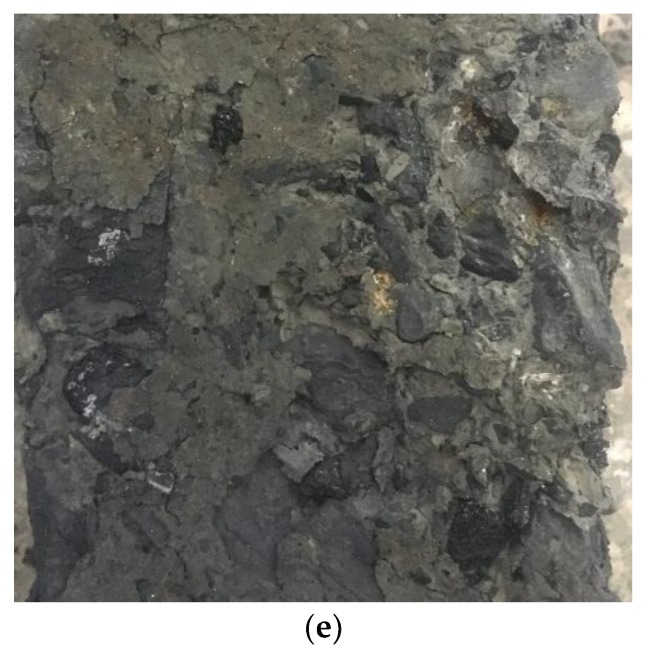
Surface pattern of I-78 specimens after immersed in different days. (**a**) Immersed for 28 days; (**b**) Immersed for 56 days; (**c**) Immersed for 84 days; (**d**) Immersed for 112 days; (**e**) Immersed for 140 days.

The change of surface state for I-78 specimen at different immersion age is shown in Figure 5. The record time was set as 28, 56, 84, 112, 140 d, which is as the same as the time of collecting mass and modulus data of samples, respectively. The surface of the specimens was gradually damaged with the increasing acid immersion time. Firstly, the damaged surface could be observed much cavities with tiny size after 28 days immersion, (Figure 5a). Then, outside mortar layer started to pill out and internal coal gangue was exposed. Additionally, the original cavities have evolved to bigger concavities, as shown as Figure 5b. Next, after immersion for 84d, many cracks occurred on the corroded surfaces, and the acid solution had permeated inside the concrete by the outer cracks (Figure 5c). Figure 5d shows the packaged mortar was peeled off, and the cracks started to extend along the bonding surface that is between coal gangue and mortar. At the final stage, more and more aggregate was exposed outside, and some coal gangue was corroded heavily by acid and was peeled off. Moreover, the cracks among bonding surfaces were also expanded, which can be seen in Figure 5e. Overall, the failure process of FGM by acid can be interpreted as acid having corroded and eaten away the outside mortar at first, exposing the interior coal gangue, at which point the exposed coal gangue was damaged and peeled off from the mortar.

#### 3.4.2. Mass and Modulus Loss Rate of the Specimens

**Figure 6:**
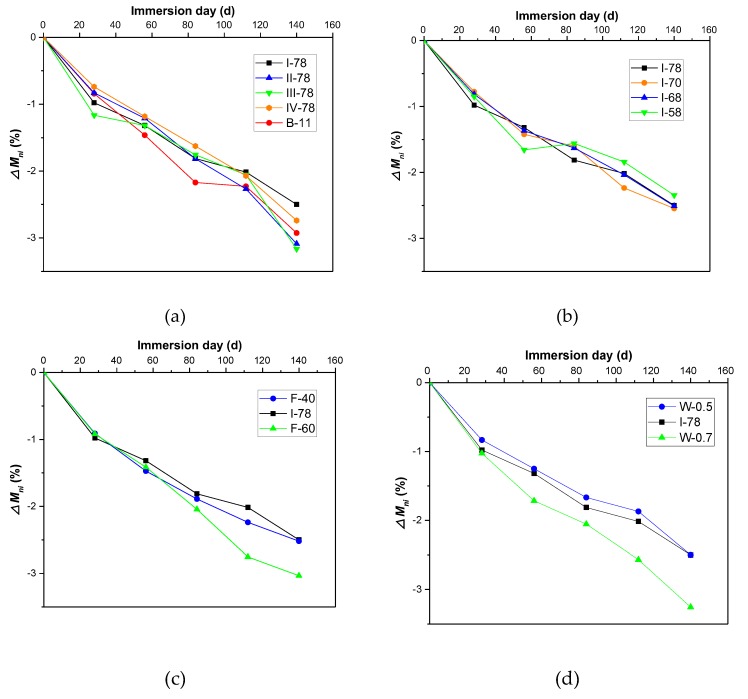
Mass loss rates of different FGM specimens in acid circumstance. (**a**) Coal gangue types and binder volume effect; (**b**) Aggregate gradation effect; (**c**) Fly ash proportion effect; (**d**) Water to binder ratio effect.

During the immersion of acid solution and water, the mass of each specimen was recorded and combined to calculate the mass loss rate, and the results are showed in Figure 6. After 140 days of corrosion, the mass loss rates were distributed over the range of 2.2–3.2%. In Figure 6a, only I-78 group had lower than 2.5% of mass-loss rate. For III-78 and II-78, this percentage could decrease to under 3%. The mixture with type I coal gangue showed a good ability in acid resistance. The coal gangue I, even used as the aggregate of the concrete, is better for durability than other coal gangues in acid circumstances. Reducing the total binder can deteriorate the ability to resist acid attack in FGM, and different aggregate gradation could not see significantly to influence the durability (Figure 6b), since the failure process was dependent on the characteristics of raw materials rather than the gradation. Figure 6c shows adding fly ash proportion from 40% to 50% could help to resist the acid attack; however, at the proportion of 60%, the mass of the specimen still dramatically decreased. Fly ash has many glass-micro ballon structures that can fill the pore of mortar. Appropriate fly ash proportion (50% of binder) could improve the microstructure of cement systems, which helps to resist corrosion of acid [49]. Over the suitable fly ash proportion of binder, the cement production will not be enough to bond with aggregate, so that the bonding interface between aggregate and mortar shows more drawbacks and cannot be more compatibility. With the water to binder ratio increasing, the specimen will be heavily damaged by acid solution, which can be found in W-0.7 group (water to binder ratio is 0.7), while W-0.5 and I-78 group cannot see many distinct differences in mass loss rate (Figure 6d). Zhou [22] did the indoor test to study the degradation characteristics of CFG pile body material in contaminated environment, of which material was ordinary used in reinforcing foundation. The CFG pile body material was made up of fly ash, gravel, stone chips, and cement, and the difference with FGM is the aggregate that originated from limestone. In his tests, the mass and the mechanical properties will also change dramatically under the condition of nitric acid erosion. After the specimens soaked in 25 g/L nitric acid solution (pH ≈ 2) for 140 days, the mass loss rate of CFG material will be at the range of 3.97–4.96% [22]. It is clear that this extent of degradation is worse than FGM (mass loss rate only below 3.5%), and FGM has the ability to keep its integrity in acid surroundings.

**Figure 7:**
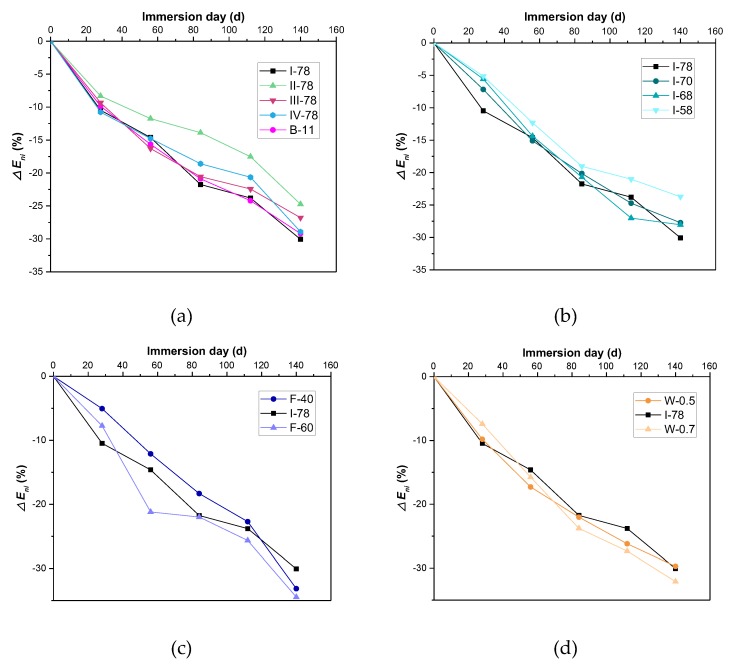
Modulus loss rate of different FGM specimens in acid circumstance. (**a**) Coal gangue types and binder volume effect; (**b**) Aggregate gradation effect; (**c**) Fly ash proportion effect; (**d**) Water to binder ratio effect.

The results of dynamic elastic modulus loss rate of different specimens are shown in Figure 7. As shown in Figure 7, the modulus rate of FGM all experienced steady decrease, and within 140 days, the loss rates could be below 35%. The value dots did not see much dispersion. In comparison with the results reported by Zhou [22], the modulus loss rate of CFG that was obtained by the same formulation as Equation (4) can reach up to 48.6%, which means deeper deterioration. Therefore, in certain cases, FGM shows the better resistance to acid attack. For CFG failure, it was made up of limestone, with the content of more than 75% [22]. Limestone belongs to alkali oxide, because its CaO proportion accounts up more than 50% [24]. Compared with Table 1, it is different from coal gangue that limestone can easily neutralize acid or be neutralized by acid. 

#### 3.4.3. Strength Loss Rate

**Figure 8:**
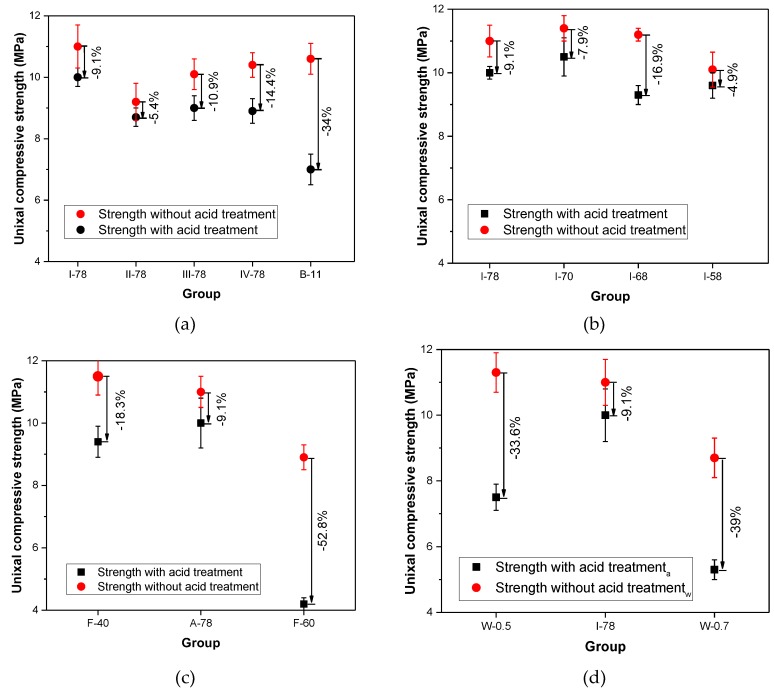
Uniaxial compressive strength of the FGM specimens immersed in acid and water. (**a**) Coal gangues types and binder volume effect; (**b**) Aggregate gradation effect; (**c**) Fly ash proportion effect; (**d**) Water to cement ratio effect.

The uniaxial compressive strength was also used as an index to evaluate the durability of acid resistance. Figure 8 shows how much strength had been lost after 140 days corrosion. The $\Delta \sigma_{\iota }$ (strength loss rate) of each group was calculated by Equation (6). In Figure 8a, the loss rate of strength were situated 5–15%. Different coal gangue could not contribute to much difference in strength loss. However, B-11 showed a dramatic decrease in strength, which illustrated that reduction of binder amount could lead to much losses in compressive strength after acid corrosion. Figure 8b shows different gradation could lead to strength reduction among the range from 4.9–16.9%. While reducing cement proportion or increasing water to cement could diminish strength by 39–52.8% (seen in Figure 8c,d). For the results of CFG [22], the strength loss rate was at the range from 14% to 44%, which could not show obvious differences with FGM in strength loss (5.4–52.8%). Considering the accident errors and the different of experiment (uniaxial strength in this study and unconfined compressive strength in the other research [22]), therefore, the law of the strength loss in acid circumstance still need more experiments to investigate. 

To design FGM that has a excellent durability, it is essential to increase the binder or cement proportion. Fly-ash can have a positive effect in alleviating acid permeation of the special content, since if there is less cement, the aggregate cannot be bound together well. Therefore, the optimum fly ash to cement ratio is around 1:1. When the w/b is more than 0.6, the FGM cannot be applied in acid surroundings because of its high mass losses (corrosion). The aggregate gradation has no significant effect on acid resistance to be observed in this study.

### 3.5. Greenness of FGM

#### 3.5.1. Indices of e-CO_2_ and e-energy

**Figure 9:**
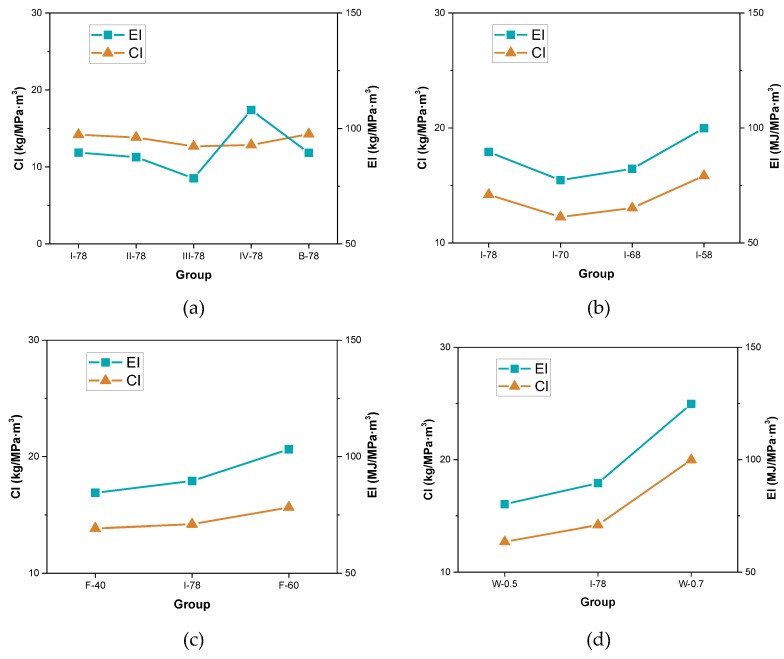
Indices of e-CO_2_ and e-energy of different FGM specimens. (**a**) Coal gangue type and binder volume effect; (**b**) Aggregate gradation effect; (**c**) Fly ash proportion effect; (**d**) Water to cement ratio effect.

The indices of CI and EI were put forward in above section to evaluate the greenness degree of FGM, and the results are given in Figure 9. From the results shown in Figure 9a, it can be seen that reduction of binder amount results in more carbon dioxide emission and more energy consumption. As far as high consumption in cement industry, reducing direct requirements of cement can cut down supplying inputs of fossil fuel and other greenhouse gas emission sources [39]. Using different coal gangues can also lead to differences in greenness degree. Coal gangue IV sees much more energy consumption, and coal gangue III shows lower CO_2_ emission. Because coal gangue from Changzhi city is more easy to be crushed and filtered by the machine, the process could spend less energy consumption, and it could have higher environmental economic value. Aggregate gradation took less environmental impacts, as the total volume of coal gangue was not changed. However, “8-18 brand” gradation improved the strength at the optimum extent, which could make the most use of the solid waste resources (see I-78 group in Figure 9b). Figure 9c shows that fly ash to cement ratio could also exist an optimal value that leads the highest greenness in FGM and the optimal ratio should be at around 0.5, and continuously increasing or decreasing the ratio will not achieve the lowest indices or good greenness. Although there are not significant energy consumption and CO_2_ emission in the production of fly ash, the cement plays a vital role in strength contribution, for FGM application. In Figure 9d, with the increase of w/b, the indices climbed more dramatically. Because the strength declined a lot in this situation, which can be found in Figure 9d. Therefore, it indicates that the production of FGM should control the water to binder ratio (no more than 0.6).

According to the results reported by Zhou [22], water-binder ratio of CFG is at the range of 0.4–0.6, the fly ash content is 40–60%, the agregate proportion is 75%. Therefore, this mix proportion is almost the same as that of FGM in this article. In addition, the e-CO_2_ and e-energy of unit m^3^ CFG were calculated and presented in Table 6. It can be found from Table 6 that the CI of CFG is among 9–15.4 kg/MPa·m^3^, which is similar with that of FGM. However, the EI indices of CFG were mostly more than 300 MJ/MPa·m^3^, which doubled than EI indices of FGM. Overall, FGM shows a better ability than traditional foundation improvement technology in saving energy consumption for preparation.

#### 3.5.2. Relationships Between Environmental Impact Indices of FGM and Compressive Strength 

**Figure 10:**
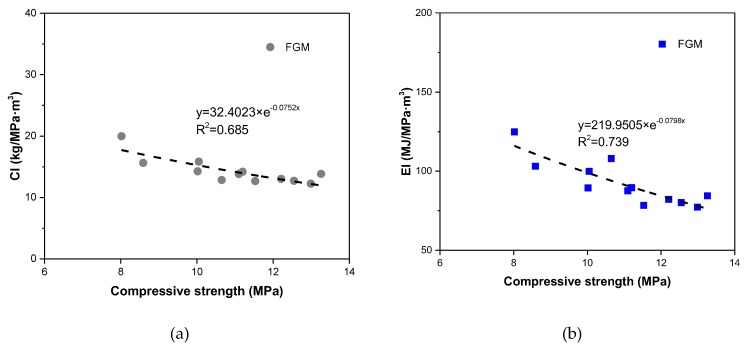
Relationship between greenness indices and compressive strength in FGM. (**a**) Relationship between CI and compressive strength in FGM; (**b**) Relationship between EI and compressive strength in FGM.

According to the above results, the relationships between compressive strength and CI, EI indicies were demonstrated in Figure 10. Two power functions can fit well with these relationships, with R^2^ coefficient approaching 0.7. In the previous research [23,24], the same relationships in self-compacting concrete and ultral high-performance concrete were also investigated. As same as the investigated law, the increase of compressive strength of FGM corresponds to a gradual decline of the e-CO_2_ index (CI) and the e-energy index (EI), whatever the mix proportion is. Consequently, it can indicate that an FGM with lower CO_2_ emissions and lower energy consumption can be achieved by designing a reasonably higher compressive strength.

#### 3.5.3. Designing High Performance and High Greenness FGM

Based on the above results, it can be found that the greenness indices, including e-CO_2_ index and e-energy index, were greatly influenced by the mixing proportion parameters. Selecting a reasonable coal gangue can help to decrease the environmental impact of FGM. For example, coal gangue III (Changzhi coal gangue) was investigated to not only reduce the carbon dioxide exhausted but also save energy for processing. Adjusting the aggregate gradation could not take effect on the environment directly, but it can help to achieve the optimal strength and also benefit for promoting sustainable developments of construction materials indirectly. Replacing certain cement content with fly-ash, as possible as avoiding cementitious materials, can also reach the optimal degree in greenness, so applying suitable fly ash to cement ratio can effectively reduce environmental impacts as expected. Although water content can improve the flowability of fresh FGM concrete, it is bad for strength development, particularly at over 0.6 of w/b. Overall, one can refer to the following suggestions about designing a more sustainable and cleaner FGM with high performance:

(1) Selection of the regional coal gangue makes high strength in FGM, such as the source from Changzhi in this experiment.
(2) Ensuring a reasonable aggregate gradation by optimizing the ratio of fine aggregate to coarse aggregate. The optimal ratio is 3:7 in this experiment.
(3) Determining a suitable ratio of fly ash to cement, the optimal ratio is around 1:1 in this study.
(4) Keeping the low ratio of water to binder. It is suggested that the ratio should be no more than 0.6.

## 4. Conclusions

It is feasible to develop a green low strength mixture FGM for foundation reinforcement treatment by using high volume of fly-ash and coal gangue aggregate, comprising more than 83% of mass. This treatment greatly reduces the consumption of cement and natural aggregates, like sand or limestone. The reasonable mix proportions for FGM material were designed and proposed on the basis of investigation on mechanical properties and durability performances and even greenness. The main conclusions are summarized as follows:

1. The factors of coal gangue types, binder amount, aggregate gradation, water to binder ratio (w/b) and fly ash proportion can greatly influence the strength and durability of FGM. Decreasing w/b or fly ash proportion in the total binder will enhance strength and durability. Larger total binder content can do so. The “8-18” band aggregate gradation is an optimal mix to design FGM with the highest strength, while it cannot deteriorate the durability.
2. Preparing FGM with below 0.6 of w/b and below 50% fly ash of total binder can product over 10 MPa strength in the material. When considering environmental influence, it should keep a certain cement content at low. Therefore, to design high durability FGM with green and cleaning, w/b should be reduced to below 0.6. Then, coal gangue I (from Xuchang) and coal gangue IV (from Yulin) are good raw materials to be selected.
3. Regardless of the FGM mix proportion, the indices of e-CO_2_ and e-energy go down with the compressive strength increasing. However, it is suitable for FGM with a compressive strength ranging from 8–14 MPa. In the comparison of previous treatments for consolidating foundation, the significant advantage of FGM material has the lower energy consumption and smaller carbon emission in unit m^3^ preparation. However, it does not show much on CO_2_ exhausting reduction from unit MPa·m^3^ in FGM.
4. Advices was provided for designing and preparing FGM with high quality and low carbon emissions. It can promote foundational technology with lower costs, greater sustainability, and an overall high environmental value.

## Figures and Tables

**Table 1:** Chemical compositions of cement, fly ash, and coal gangues.

Oxide	Cement	Fly ash	Coal Gangue I	Coal Gangue II	Coal Gangue III	Coal Gangue IV
	Percent (%)					
SiO_2_	22.37	53.10	57.71	60.19	61.05	65.87
Al_2_O_3_	4.36	2.93	28.64	29.28	25.92	20.25
Fe_2_O_3_	3.38	10.20	4.66	3.25	3.06	4.59
CaO	61.08	21.80	2.24	1.15	3.07	0.45
MgO	2.43	-	0.61	0.71	0.83	1.77
SO_3_	2.45	0.58	0.80	0.44	0.20	0.20
Na_2_O_eq_	0.51	-	0.58	0.21	0.61	1.98
K_2_O	-	-	2.99	3.07	3.67	3.98
IL	1.33	5.83	-	-	-	-

**Table 2:** Mix proportions for FGM specimens.

Serials	Cement kg/m^3^	Fly Ash kg/m^3^	Coal Gangue (kg/m^3^)		Water kg/m^3^	Coal Gangue Type	e-CO_2_ (kg/m^3^)	e-energy (MJ/m^3^)
			0–4.75 mm	4.75–31.5 mm				
I-78	150	150	400	1440	180	I	158.97	1003
II-78	150	150	400	1440	180	II	153.45	972
III-78	150	150	400	1440	180	III	146.09	904
IV-78	150	150	400	1440	180	IV	136.89	1150
B-11	130	130	420	1460	180	I	142.91	896
I-70	150	150	560	1280	180	I	158.97	1003
I-68	150	150	590	1250	180	I	158.97	1003
I-58	150	150	780	1060	180	I	158.97	1003
F-40	180	120	400	1440	180	I	183.60	1120
F-60	120	180	400	1440	180	I	134.34	886
W-0.5	150	150	415	1455	150	I	159.51	1006
W-0.7	150	150	485	1425	210	I	160.23	1001

I-number groups have various aggregate gradation respectively; B-11 group has the lower volume of binder (11%), and other groups uniformly have binder agent with 13% of total mass; F-number groups have different fly-ash proportion in total binder; W-number groups have different water to binder ratios (w/b).

**Table 3:** Embodied carbon dioxide (e-CO_2_) and embodied energy of the raw materials in FGM.

Items	e-CO_2_	e-energy	References
Cement	0.83	4.727	[23,24]
Fly ash	0.009	0.833	[23,24]
Coal gangue I	0.018	0.092	[38]
Coal gangue II	0.015	0.075	[38]
Coal gangue III	0.011	0.038	[38]
Coal gangue IV	0.006	0.172	[38]
Limestone	0.041	3.9	[39,40]

**Table 4:** Dynamic modulus and Poisson’s ratio results of four series of mixtures.

No. of Specimen	Curing Ages (d)	Dynamic Electricity Modulus (GPa)	Dynamic Shear Modulus (GPa)	Poisson’s Ratio
I-78	28	14.8	4.9	0.510
II-78	28	11.2	3.5	0.600
III-78	28	10.5	3.4	0.544
IV-78	28	10.7	3.5	0.528
B-11	28	14.7	4.1	0.787
I-70	28	15.2	5.2	0.472
I-68	28	15.1	5.1	0.492
I-58	28	14.2	4.6	0.542
F-40	28	16.4	5.3	0.536
F-60	28	14.3	4.6	0.552
W-0.5	28	16.3	5.3	0.527
W-0.7	28	13.2	4.5	0.478
I-78	60	16.2	5.3	0.528
II-78	60	13.2	4.2	0.581
III-78	60	12.7	4.3	0.485
IV-78	60	12.8	4.1	0.561
B-11	60	15.1	4.2	0.779
I-70	60	16.3	5.6	0.460
I-68	60	16.0	5.3	0.499
I-58	60	15.2	4.9	0.541
F-40	60	17.8	5.8	0.542
F-60	60	15.6	5.1	0.544
W-0.5	60	17.7	5.8	0.532
W-0.7	60	14.6	4.9	0.483

**Table 5:** Fitting parameters of the stress–strain curve for different specimens.

No. of Specimen	Ascending Segment		Descending Segment	
	a	R^2^	b	R^2^
I-78	−1.14	0.98	12.79	0.99
II-78	−0.77	0.99	8.85	0.99
III-78	−0.90	0.98	11.19	0.97
IV-78	0.49	0.99	9.35	0.99
B-11	−0.61	0.98	13.96	0.99
I-70	−1.88	0.94	30.46	0.99
I-68	−1.40	0.99	27.55	0.99
I-58	−0.04	0.99	5.99	0.98
F-40	−0.69	0.99	22.74	0.99
F-60	−0.88	0.99	4.91	0.98
W-0.5	−1.12	0.97	15.86	0.98
W-0.7	−1.31	0.99	7.71	0.98

**Table 6:** Greenness indices of CFG.

Serials	e-CO_2_ (kg/m^3^) [22]	e-energy (MJ/m^3^) [22]	28-Day Strength [22]	CI (kg/MPa·m^3^)	EI (MJ/MPa·m^3^)
S1/F1	233.5	7860.9	17.9	13.0	437.4
S2	265.3	8011.5	21.8	12.2	367.7
S3	201.8	7710.4	13.1	15.4	588.6
F2	237.5	7994.2	26.5	9.0	302.1
F3	229.7	7732.1	18.1	12.7	428.4
